# ALSFRS-R-SE: an adapted, annotated, and self-explanatory version of the revised amyotrophic lateral sclerosis functional rating scale

**DOI:** 10.1186/s42466-022-00224-6

**Published:** 2022-12-15

**Authors:** André Maier, Matthias Boentert, Peter Reilich, Simon Witzel, Susanne Petri, Julian Großkreutz, Moritz Metelmann, Paul Lingor, Isabell Cordts, Johannes Dorst, Daniel Zeller, René Günther, Tim Hagenacker, Torsten Grehl, Susanne Spittel, Joachim Schuster, Albert Ludolph, Thomas Meyer, for the MND-NET consensus group

**Affiliations:** 1 Department of Neurology, Center for ALS and Other Motor Neuron Disorders, Charité – Universitätsmedizin Berlin, Corporate Member of Freie Universität Berlin, Humboldt-Universität zu Berlin, and Berlin Institute of Health, Berlin, Germany; 2 Department of Neurology, Universitätsklinikum Münster, Münster, Germany; 3 Department of Medicine, UKM-Marienhospital Steinfurt, Steinfurt, Germany; 4 Friedrich-Baur-Institut und Neurologische Klinik und Poliklinik, LMU Klinikum, Ludwig-Maximilians-Universität München, Munich, Germany; 5 Klinik für Neurologie, Universitätsklinikum Ulm, Ulm, Germany; 6 Hannover Medical School, Department of Neurology, Hannover, Germany; 7 Department of Neurology, Campus Lübeck, Universitätsmedizin Schleswig-Holstein, Lübeck, Germany; 8 Department of Neurology, Universitätsklinikum Leipzig, Leipzig, Germany; 9 Department of Neurology, Klinikum rechts der Isar, Technical University Munich, Munich, Germany; 10 Department of Neurology, Universitätsklinikum Würzburg, Würzburg, Germany; 11 Department of Neurology, University Hospital Carl Gustav Carus, Technische Universität Dresden, Dresden, Germany; 12 DZNE, German Center for Neurodegenerative Diseases, Research Site Dresden, Dresden, Germany; 13 Klinik für Neurologie und Center for Translational Neuro- and Behavioral Science, Universitätsmedizin Essen, Essen, Germany; 14 Department of Neurology, Centre for ALS and Other Motor Neuron Disorders, Alfried Krupp Krankenhaus, Essen, Germany; 15 Ambulanzpartner Soziotechnologie APST GmbH, Berlin, Germany; 16 DZNE, German Centre for Neurodegenerative Diseases, Research Site Ulm, Ulm, Germany

**Keywords:** Amyotrophic lateral sclerosis, ALSFRS-R, Patient reported outcomes, Digital medicine

## Abstract

**Background:**

The ALS Functional Rating Scale in its revised version (ALSFRS-R) is a disease-specific severity score that reflects motor impairment and functional deterioration in people with amyotrophic lateral sclerosis (ALS). It has been widely applied in both clinical practice and ALS research. However, in Germany, several variants of the scale, each differing slightly from the others, have developed over time and are currently in circulation. This lack of uniformity potentially hampers data interpretation and may decrease item validity. Furthermore, shortcomings within the standard ALSFRS-R questions and answer options can limit the quality and conclusiveness of collected data.

**Methods:**

In a multistage consensus-building process, 18 clinical ALS experts from the German ALS/MND network analyzed the ALSFRS-R in its current form and created an adapted, annotated, and revised scale that closely adheres to the well-established standardized English version.

**Results:**

Ten German-language variants of the ALSFRS-R were collected, three of which contained instructions for self-assessment. All of these variants were compiled and a comprehensive linguistic revision was undertaken. A short introduction was added to the resulting scale, comprising general instructions for use and explanations for each of the five reply options per item. This adapted version of the scale, named ALSFRS-R-SE (with the “SE” referring to “self-explanatory”), was carefully reviewed for language and comprehensibility, in both German and English.

**Conclusion:**

An adapted and annotated version of the ALSFRS-R scale was developed through a multistage consensus process. The decision to include brief explanations of specific scale items and reply options was intended to facilitate ALSFRS-R-SE assessments by both healthcare professionals and patients. Further studies are required to investigate the accuracy and utility of the ALSFRS-R-SE in controlled trials and clinical real-world settings.

**Supplementary Information:**

The online version contains supplementary material available at 10.1186/s42466-022-00224-6.

## Introduction

The Amyotrophic Lateral Sclerosis Rating Scale in its revised version (ALSFRS-R) [1] is the most widely used instrument for assessing functional deficits in amyotrophic lateral sclerosis (ALS) [2]. The scale is disease-specific and encompasses 12 prompts-referred to as items-grouped into four domains to assess bulbar symptoms, limb and trunk functionality, respiratory symptoms, and the need for percutaneous endoscopic gastrostomy, non-invasive ventilation, or tracheostomy with invasive ventilation [1]. The precursor scale was initially developed as an outcome measure for clinical trials [3], but over time its revised version became commonly used in both ALS research and clinical practice [4–6]. Assessments were originally conducted exclusively through in-person and telephone interviews with healthcare professionals [4, 7], but self-assessments and online questionnaires are now part of standard practice [8, 9].

Functional assessment via the ALSFRS-R is one of the most significant outcomes in clinical ALS trials, both because surrogate parameters such as neurofilaments have only recently become available and because this method is accepted for determining clinically meaningful outcomes [10]. In addition, the scale allows for modeling of individual disease courses [11, 12], can predict survival [13], and supports the staging of ALS as done with Milano-Torino (MiToS) functional staging or the King's system [14]. Although the second is not based on the ALSFRS-R score, it can be deducted from it with 92% concordance [15]. Since cognitive and executive deficits occur within the ALS spectrum and the disease pathophysiologically overlaps with frontotemporal degeneration, functional assessments in studies are often complemented by questionnaire-based cognitive tests, e.g., the Edinburgh Cognitive and Behavioural ALS Screen (ECAS) [16]. The further inclusion of disease-specific quality of life instruments such as the ALS Assessment Questionnaire ALSAQ-40 [17], or more generic tool like the EuroQol 5 Dimension (EQ-5D) [18], contribute to a comprehensive approach to ALS research.

Although the ALSFRS-R is a validated instrument with high inter-rater and intra-rater reliability [4, 9, 19], design inconsistencies have been reported in both the description and classification of items in the ALSFRS-R [20, 21]. Recent observations revealed substantial deviations in consecutive assessments [22] and inconsistent application of the scale, particularly in the area of respiration [23]. Due to a lack of consistent standard operating procedures, the original ALSFRS-R may not adequately represent functional deficits [24]. Close analysis of the scale suggests that the original ALSFRS-R falls short of meeting all the conceptual requirements placed upon it [25, 26]. Furthermore, several variants of the same scale have evolved and circulated over time in Germany, at least. The existence of scale variants may be explained by the absence of a validated German-language version of the ALSFRS-R. As a result, translation of the ALSFRS-R has primarily taken place at the initiative of individual institutions. Alternately, researchers often use a shortened form of the ALSFRS-EX. This extended scale is a version of a self-assessment test, available in German, with three items added [27]. While the ALSFRS-EX mitigates the floor effect associated with the ALSFRS-R score, it falls short of resolving the fundamental issues in need of addressing.

Consequently, there have been calls not only in Germany but around the world to refine, harmonize, and revise the ALSFRS-R in terms of language accuracy [22, 24, 25]. As it is used as an endpoint in clinical trials and plays a crucial role in monitoring disease progression in routine care for individuals with ALS, overcoming the scale’s methodological shortcomings is paramount to its continued use in the future.

Raters who participate in clinical trials typically undergo ALSFRS-R training based on the guidelines of different certifying organizations; namely those of the Northeast ALS Consortium (NEALS) in association with the Barrow Neurological Institute (BNI), or the European Network for the Cure of ALS (ENCALS). However, demand for an internationally consistent ALSFRS-R scale has been tempered by divergent training content and the observation that raters’ skills may decrease over time [22]. Another factor that might further comprise the quality of data assessment is the lack of available ALSFRS-R training in languages other than English. To compensate for any ambiguity surrounding the description of individual items and reply options within the scale, and to reduce training requirements for conducting assessments, explanatory comments have already been added to several version of the scale [19, 27, 28].

## Methods

### Definition of the terms “version” and “variant”

Over the course of developing an optimized version of the ALSFRS-R, it became evident that the terms “version” and “variant” have to be distinguished. An ALSFRS-R “version” is defined relative to its place in the evolution of the scale. The predecessor of ALSFRS-R, for example, is the ALSFRS version. Subsequent versions of the ALSFRS-R include the ALSFRS-EX and validated translations in various languages [29–37] (which raise previously mentioned problems of interpretation). In contrast, “variants” are slight deviations that occur within the original scale. For example, variants may emerge as the result of parallel translations, adaptations by different institutions, or simply through truncation.

### Consensus building

**Fig. 1:**
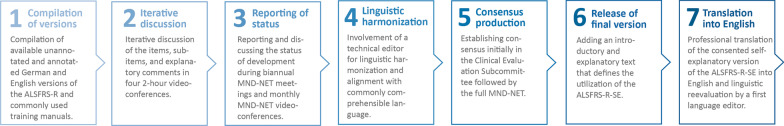
seven-step consensus process to develop the ALSFRS-R-SE

A seven-step consensus process with the aim of optimizing the ALSFRS-R was carried out between October 2020 and December 2021 (Fig. 1). The initiative was conducted by members of the “German ALS/MND-NET” – a clinical and scientific network of ALS/motor neuron disease centers with 27 sites in Germany, two partners in Switzerland and one partner in Austria. This consensus group encompassed 18 ALS experts, each with long-standing expertise in ALSFRS-R assessments and ALSFRS-R certifications from at least one, and most often two, organizations.

For linguistic revision and translation into English, a technical editor, a professional translator, and first language editor were brought in, neither of whom have backgrounds in ALS.

### Design

A consensus group design [38] was used to develop an optimized version of the ALSFRS-R scale. To assess the existing ALSFRS-R variants in circulation, more than 120 neurologists, researchers, and study nurses within the MND-NET were contacted via e-mail and asked to share the German-language ALSFRS-R forms that they were using at the time.

### Preconditions

Optimizing the ALSFRS-R scale to closely adhere to the standard English-language version [1] took the following factors into consideration: (1) Since the assessment process conducted by healthcare professionals and the self-assessments performed by patients and patient caregivers are well established, the ALSFRS-R should be accessible to both healthcare professionals and patients. In order to facilitate this, there should be no ambiguity surrounding the language of the individual diagnostic items. (2) Adding explanatory language to the scale items and reply options may reduce ambiguity as well as the need for ALSFRS-R training. (3) As ALSFRS-R assessments through computers, mobile devices, and other remote digital means are increasingly common, it is important to consider the scale’s use and suitability for these media.

## Results

### Status quo of German ALSFRS-R variants currently in use and the process towards consensus

**Fig. 2:**
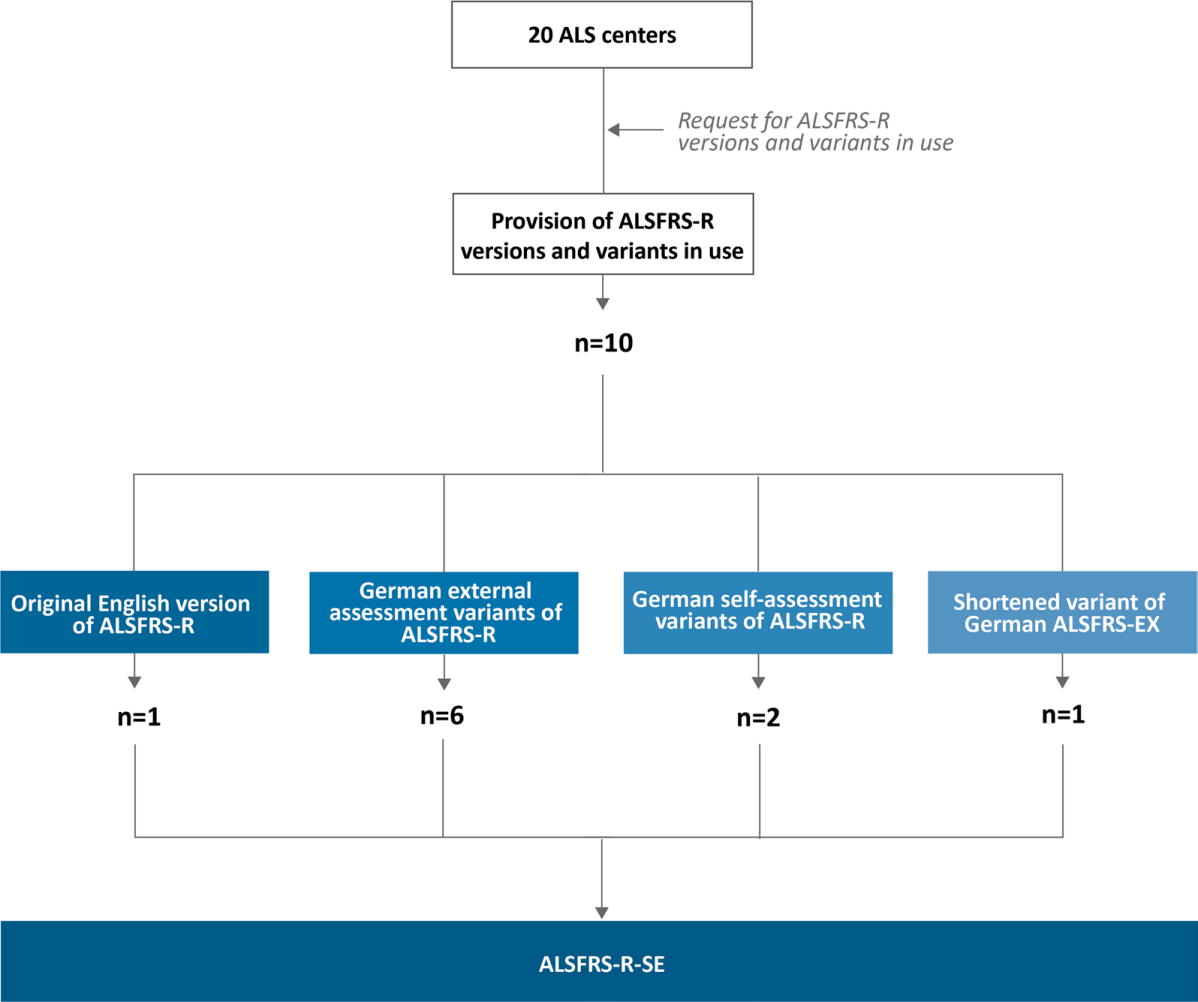
Overview of origins of ALSFRS-R versions and variants and the collection process. A total of 20-ALS centers responded to the request to provide extant ALSFRS-R versions and variants. Nine different German variants and the original English version by Cedarbaum et al. [1] were collected. Translated variants differed in terms of wording. Two variants were designated for self-assessment. Several ALS centers use a shortened variant of the ALSFRS-EX [27]

Nine German language variants of the ALSFRS-R were collected, three of which already contained instructions for self-assessment. Six variants were individual translations of the original English version for external evaluation. Figure 2 depicts the schematic process of consolidating the pre-existing English scale and nine German variants of the ALSFRS-R. The additional files includes the German and English-language versions of the ALSFRS-R-SE that emerged from the consensus process [see Additional file 1 and Additional file 2].

### Item structure of the ALSFRS-R and impact of assistive measures

The ALSFRS-R is a functional scale that measures deviations from unrestricted or "normal" motor functioning as caused by ALS. The two major symptoms that present in ALS and limit functionality in specific areas are muscle weakness and stiffness. Muscle weakness is caused predominantly by the loss of the second motor neuron. Muscle stiffness often, but not always, results from spasticity, indicating degeneration of the upper neuron [39]. If possible, complaints that are not related to ALS-such as orthopedic issues that might affect functional areas-should be excluded from the evaluation. There are two ways to do this: Firstly, functionality should always be assessed in relation to the person’s status before the onset of ALS symptoms; and secondly, conditions that are obviously unrelated to ALS should be excluded from the assessment even if they lead to functional impairment.

To ordinally scale the loss of functionality, anchor points grade functionality from 0 to 4 for each item. To determine an ordinal score, the scale considers whether there is an increased need for assistance or assistive devices. The most unambiguous scores are 4, which designates unrestricted functionality, and 0, which designates a complete loss of functionality. Whereas the latter score can be identified by the full-time use of assistive procedures or devices, differentiating between intermediary scores can be difficult. A mild impairment (score 3) should reflect a condition that does not yet require compensatory help. Score 2 is characterized by intermittent use of compensatory measures. Score 1 is given if assistive procedures or devices are needed in all instances, and independence is severely reduced but not entirely lost. These considerations apply to the first nine items and to item 11. With regard to respiratory items 10 and 12 functional compensation (i.e., mechanical ventilation) is implied either at the 0-point level (item 10: dyspnea) or already at the 3-point level (item 12: respiratory insufficiency). This change does not eliminate the structure of those items, but instead shifts the anchor points in a given direction.

The specific moment in which personal assistance, technical support, or other substituting procedures are first used may be influenced by several factors, including the personal convictions of the affected person and the availability of assistive options such as PEG or mechanical ventilation. Since circumstantial considerations do not necessarily reflect an affected person’s functional capacity, they should not overrule the implicit logic of an item.

### Explanatory introduction

To illustrate the operation of the ALSFRS-R and to standardize its application, an explanatory introduction was included (see bottom of Table 1). The introduction instructs any individual using the ALSFRS-R-health care professional, family member, or a patient-to carefully review the response options and their corresponding comments and to select the most applicable answer. With regard to pre-existing functional limitations, the recommendation is to score the item as “normal” or "unrestricted" by ALS (4 points). This recommendation should be waived only if ALS symptoms have exacerbated a pre-existing impairment. Once an approach is chosen, it should be followed consistently across all items and in future ALSFRS-R assessments.

**Table 1:** ALSFRS-R versus ALSFRS-R-SE after consensus process

No/Pts	ALSFRS-R item	ALSFRS-R-SE item	ALSFRS-R-SE item additional explanation	Type of adaption of ALSFRS-R-SE	Deviation from ALSFRS-R
1	*Speech*	*Speech*			
1.4	Normal speech process	Normal	Speaking and/or articulation is the same as it was before the onset of initial ALS symptoms	Harmonization	Minor
1.3	Detectable speech disturbance	Detectable speech disturbance	Speech, articulation or phonation have changed, as perceived either by the affected themselves or by their immediate contacts. This may present as slurred speech or hoarseness of voice	None	
1.2	Intelligible with repeating	Intelligible speech with repetition	Frequent repetition of single words or parts of a sentence are required to convey meaning	Linguistic adaption	Minor
1.1	Speech combined with non-vocal communication	Speech combined with non-vocal communication	Writing things down, use of communication aids, and similar methods are needed to convey meaning	None	
1.0	Loss of useful speech	Loss of useful speech	Communication aids or similar methods are always required	None	
2	*Salivation*	*Salivation*			
2.4	Normal	Normal	No excess saliva accumulates in the mouth	None	
2.3	Slight but definite excess of saliva in mouth; may have night-time drooling	Slight but definite excess of saliva in mouth; night-time drooling may take place	Increased accumulation of saliva in the mouth; however, subjectively not an impairment or impediment and no loss of saliva during the day	Linguistic adaption	Minor
2.2	Moderately excessive saliva; may have minimal drooling	Moderately excessive saliva; may experience minimal drooling	During the day, a tissue is occasionally used to dab the edges of the mouth	Linguistic adaption	Minor
2.1	Marked excess of saliva with some drooling	Marked excess of saliva with some drooling	Regular loss of saliva, a tissue is used often but not constantly	None	
2.0	Marked drooling	Marked drooling	Permanent use of tissues or a suction device is required	None	
3	*Swallowing*	*Swallowing*			
3.4	Normal eating habits	Normal	Swallowing any type of food or liquid is unproblematic	Harmonization	Minor
3.3	Early eating problems-occasional choking	Minor swallowing problems-occasional choking	Food intake takes longer; food must be cut into smaller bites and swallowed with care. Occasionally, choking on food or higher frequency of coughing is observed	Linguistic adaption	Minor
3.2	Dietary consistency changes	Dietary consistency changes	Difficulty swallowing (dysphagia) and trouble with certain consistencies of food and beverages results in the avoidance of some types of food consistencies (e.g., meat, dry biscuits, nuts). Dietary supplements or thickeners may be used due to difficulty in swallowing	None	
3.1	Needs supplemental tube feeding	Supplemental tube feeding	Due to dysphagia, food intake has become so difficult that an enteral feeding tube (PEG) must be fitted or is highly recommended by the physician to supplement caloric intake and/or prevent choking on food	Harmonization	Minor
3.0	NPO (exclusively parenteral or enteral feeding)	Exclusively enteral tube feeding	Food and liquid intake happen exclusively via a feeding tube; oral food intake is impossible due to high-grade dysphagia	Linguistic adaption and harmonization	Moderate
4	*Handwriting*	*Handwriting*	The subject of this assessment is writing with one’s dominant hand (writing hand) in the usual posture	Addition	Moderate
4.4	Normal	Normal	Writing with the dominant writing hand causes no problems	None	
4.3	Slow or sloppy: all words are legible	Slow or sloppy, but all words are legible	Writing is more difficult, or alternately, the appearance of a person's written text has changed even though the words remain legible	Harmonization	Minor
4.2	Not all words are legible	Not all words are legible	Some written words are illegible. Writing aids are used to promote legibility	None	
4.1	Able to grip pen but unable to write	Able to grip pen but unable to write	Holding a pen is possible; however, anything beyond signing or writing one's own name is not	None	
4.0	Unable to grip pen	Unable to grip pen	Holding a pen is impossible	None	
5a	*Cutting food and handling utensils*	*Cutting food and handling utensils*	Pertaining to persons not regularly using an enteral feeding tube for caloric intake	Addition	Moderate
5a.4	Normal	Normal	The use of cutlery is not problematic. Problems would be, for example, the use of knives and forks instead of chopsticks or the inclination to use a spoon more often	None	
5a.3	Somewhat slow and clumsy, but no help needed	Somewhat slow and clumsy, but no help needed	Eating takes more time due to impairment of the hands. Method of handing cutlery has changed, but its use is still possible without assistance	None	
5a.2	Can cut most foods, although clumsy and slow; some help needed	Can cut most foods, although slowly and clumsily; some help is needed	Assistance is needed on occasion when cutting certain types of foods; alternately, eating aids such as special cutlery are in use	Linguistic adaption	Minor
5a.1	Food must be cut by someone, but can still feed slowly	Food must be cut by someone else, but can still feed themself slowly	Assistance is needed to cut solid food and on most other occasions. However, eating on one's own is still possible (e.g., using a fork or a spoon)	Linguistic adaption	Minor
5a.0	Needs to be fed	Total dependence	The affected person is unable to use cutlery (e.g., a fork or a spoon) on their own, and can only eat when fed	Harmonization	Moderate
5b	*Cutting food and handling utensils*	*Cutting food and handling utensils*	For persons regularly in need of a feeding tube for caloric intake. The subject of this assessment is manual dexterity	addition	Moderate
5b.4	Normal	Normal	The tube can be handled independently, and locks and packets can be opened and closed without assistance	None	
5b.3	Clumsy but able to perform all manipulations independently	Clumsy but able to perform all manipulations independently	No assistance is needed when handling the feeding tube, however use is somewhat difficult	None	
5b.2	Some help needed with closures and fasteners	Some help needed with closures and fasteners	Handling the feeding tube is done more or less independently. Assistance is needed when opening locks and fasteners	None	
5b.1	Provides minimal assistance to caregiver	Provides minimal assistance to caregiver	Another person mostly handles the feeding tube. The affected can only carry out minimal actions themselves	None	
5b.0	Unable to perform any aspect of a given task	Total dependence	Handling the feeding tube is done entirely by another person. No actions can be executed by the affected	Harmonization	Moderate
6	*Dressing and hygiene*	*Dressing and hygiene*			
6.4	Normal function	Normal	Getting dressed and tending to personal hygiene are unproblematic	Harmonization	
6.3	Independent and complete self-care with effort or decreased efficiency	Independent and complete self-care requires effort and is less efficient	Getting (un)dressed and tending to personal hygiene are executed more slowly than before but are performed autonomously and require neither aids nor assistance from another person	Linguistic adaption	Minor
6.2	Intermittent assistance or substitute methods	Intermittent assistance or substitute methods	At times, another person is called upon to assist, or strategies are developed to counteract impairment (e.g., wearing clothes that are easy to put on or take off, getting (un)dressed or showering while sitting down, use of aids)	None	
6.1	Attendant needed to assist with self-care	Attendant needed to assist with self-care	Another person is required on a regular basis to (un)dress and attend to the affected person’s personal hygiene	None	
6.0	Total dependence	Total dependence	Dressing, undressing and personal hygiene must be entirely performed by another person	None	
7	*Turning in bed and adjusting bed clothes*	*Turning in bed and adjusting bed clothes*			
7.4	Normal	Normal	Turning in bed and handling blankets do not cause problems	None	
7.3	Somewhat slow and clumsy, but no help needed	Somewhat slow and clumsy, but no help needed	Turning in bed or handling blankets is difficult	None	
7.2	Can turn alone or adjust sheets, but with great difficulty	Can turn on their own or adjust sheets, but with great difficulty	Turning in bed and handling blankets is possible but requires great effort. Either action may require support, or a grip may be used when turning in bed	Linguistic adaption	Minor
7.1	Can initiate, but not turn or adjust sheets alone	Can initiate action, but not turn or adjust sheets without assistance	The actions of turning in bed and handling blankets can be initiated, however another person's assistance is required to complete these actions	Linguistic adaption	Minor
7.0	Helpless	Total dependence	Assistance is consistently required when turning in bed or handling blankets	Harmonization	Moderate
8	*Walking*	*Walking*			
8.4	Normal	Normal	No change in walking ability	None	
8.3	Early ambulatory difficulties	Minor ambulatory difficulties	Changes, such as walking more slowly, stumbling, or a loss of stability, are apparent, although the affected does not require outside assistance on a regular basis, either in the form of another person, a walking aid (e.g., foot lifter, cane, walkers) or holding on to a stable object	Linguisitic adaption	
8.2	Walks with assistance	Walks with assistance	The affected regularly requires assistance when walking-either in the form of holding on to something, or, outside the home, use of a foot lifter, walking aids or help from another person	None	
8.1	Nonambulatory functional movement	Nonambulatory functional movement	Targeted leg movements are still possible. Standing with support, e.g., for transfer, can be possible. The affected has no ambulatory capacity, not even with the assistance of another person	None	
8.0	No purposeful leg movement	No purposeful leg movement	The legs cannot support the weight of the body (e.g., for transfer), no purposeful movements can be executed, such as helping with care activities	None	
9	*Climbing stairs*	*Climbing stairs*			
9.4	Normal	Normal	No change is observed when climbing the stairs	None	
9.3	Slow	Slow	Climbing the stairs without taking a break or feeling unstable is possible if done slowly	None	
9.2	Mild unsteadiness or fatigue	Mild unsteadiness or fatigue	Climbing the stairs is accompanied by a feeling of instability, and breaks might be necessary. Use of a handrail or assistance from another person are not absolutely necessary	None	
9.1	Needs assistance	Needs assistance	Climbing the stairs cannot be executed without use of a handrail or assistance from another person	None	
9.0	Cannot do	Cannot do	Stairs cannot be climbed, even with assistance or support	None	
10	*Dyspnea*	*Dyspnea and shortness of breath*		Addition	Minor
10.4	None	None	No dyspnea or shortness of breath when performing daily routines at normal intensity	None	
10.3	Occurs when walking	Occurs when walking	Dyspnea or shortness of breath may occur when walking at a normal pace or performing activities at moderate intensity	None	
10.2	Occurs during one or more of the following: eating, bathing, dressing (ADL)	Occurs during one or more of the following: eating, bathing, dressing (ADL)	Dyspnea or shortness of breath may occur when performing activities at low intensity or when talking for longer periods of time	None	
10.1	Occurs at rest, difficulty breathing when either sitting or lying	Difficulty breathing when at rest, including sitting or lying down	Dyspnea or shortness of breath in the absence of any physical strain when either sitting and/or lying down	Linguistic adaption	Minor
10.0	Significant difficulty, considering using mechanical respiratory support	Significant difficulty breathing, mechanical respiratory support may be needed	Significant dyspnea or shortness of breath is present when at rest; mask ventilation (non-invasive ventilation) or ventilation via tracheostomy must be applied to alleviate dyspnea and shortness of breath	Linguistic adaption	Moderate
11	*Orthopnea*	*Sleep disturbance due to breathing problems*	If mechanical ventilation is usually provided during the night, but sleep is possible without it, nighttime breathing should be assessed without the use of ventilation	Harmonization and Addition	Substantial
11.4	None	None	Falling asleep and sleeping through the night are unimpaired by dyspnea or shortness of breath	None	
11.3	Some difficulty sleeping at night due to shortness of breath, does not routinely use more than two pillows	Some difficulty sleeping at night due to shortness of breath, more than two pillows are not routinely used	Dyspnea and shortness of breath are present at night and when lying down. Breathing may be improved by sleeping on one side. To support the torso, a maximum of two pillows are used or the head section of the bed may be elevated by no more than 30 degrees	Linguistic adaption	Minor
11.2	Needs extra pillows in order to sleep (more than two)	More than two pillows are needed in order to sleep	When lying down flat on one's back, breathing is noticeably bothersome, which in turn disturbs the process of falling asleep and sleeping through the night. To support the torso, three or more pillows are used or the head section of the bed is elevated by more than 30 degrees	Linguistic adaption	Minor
11.1	Can only sleep sitting up	Can only sleep sitting up	A seated position must be assumed, either in bed or on a chair, to sleep	None	
11.0	Unable to sleep	Unable to sleep	Due to dyspnea or shortness of breath, sleep is impossible without mask ventilation (non-invasive ventilation) or ventilation via tracheostomy. Mechanical ventilation is in regular use to alleviate symptoms	None	
12	*Respiratory insufficiency*	*Mechanical ventilation*		Linguistic adaption	Moderate
12.4	None	None	Breathing is always an autonomous action, not requiring use of mechanical ventilation. Nocturnal air pressure support (i.e., CPAP therapy to treat sleep apnea syndrome) does not constitute mechanical ventilation	None	
12.3	Intermittent use of BiPAP	Intermittent use of non-invasive ventilation	Mask ventilation (non-invasive ventilation, e.g., BiPAP) is in use at irregular intervals or for a shorter period of time than the normal nocturnal sleep cycle	Harmonization	Minor
12.2	Continuous use of BiPAP during the night	Continuous use of non-invasive ventilation during the night	Mask ventilation (non-invasive ventilation) is in regular use at night and possibly on an hourly basis during the day (a total of 8 to 22 h in any 24-h cycle)	Harmonization	Minor
12.1	Continuous use of BiPAP during the night and day	Continuous use of non-invasive ventilation during the night and day	Mask ventilation (non-invasive ventilation) is in use almost all of the time (more than 22 h per in any 24-h cycle)	Harmonization	Minor
12.0	Invasive mechanical ventilation by intubation or tracheostomy	Invasive mechanical ventilation by intubation or tracheostomy	Continuous mechanical ventilation via a ventilation tube (intubation) or tracheostomy	None	

**Introduction:** The self-explanatory Amyotrophic Lateral Sclerosis Functional Rating Scale – Revised (ALSFRS-R-SE) is comprised of various motor functioning items that typically have limiting characteristics in ALS. The ALSFRS-R-SE assessment can be performed by patients themselves as well as by others (e.g., an attending physician, a relative, a healthcare professional) following an interview with the affected. Please carefully read the explanations and options and provide an assessment of functionality, and, respectively, relevant ALS-related limitations that reflect actual capacities at the time of filling in the questionnaire

If the cause of a limitation in any functional area is attributable to a medical condition other than ALS, or if a limitation was already present before the onset of ALS (e.g., gait impairment following hip replacement surgery) the respective item can be assessed as “normal” (4 score points). Functionality should always be assessed relative to one’s status before the onset of initial ALS symptoms. The affected may deviate from this recommendation if they are experiencing additional limitations that are likely to be due to ALS. Once an approach is chosen, please be consistent in following it when answering all questions on this and on future ALSFRS-R-SE assessments

### Revision of items

Table 1 compares the revised items in the ALSFRS-R-SE to those in the original ALSFRS-R scale. All changes are classified and evaluated. Adjustments made to individual items within the four scoring domains are described in the following sections. The wording of the English and German scales may not be perfectly identical due to linguistic differences.

### Bulbar function (Items 1–3)

*Item 1 (Speech)*: In contrast to the frequent use of the word "language" in the German variants, this discrepancy was balanced by consistent use of the term "speech." In the explanatory notes, "speech" was further subdivided into "phonation" and "articulation” in order to cover these both aspects of speech production. *Item 2 (Salivation)*: Adverbs such as "occasionally" or "often" are used to describe the frequency of an occurrence. This also applies to saliva collection and its associated methods, such as use of a tissue. *Item 3 (Swallowing)*: To more precisely describe this item, explanations were augmented to include descriptions such as "more careful swallowing" or "smaller bites." The reference to an "early eating problem" was replaced by the more specific "minor swallowing problem." Differences between various kinds of food consistencies were clarified with examples. Language concerning the use of an enteral feeding tube was explicitly contextualized in terms of dysphagia and explained in general terms.

### Fine motor function (Items 4–6)

*Item 4 (Handwriting)*: An explanation appended to this item clarifies that it refers to the dominant (writing) hand in the usual posture. The use of writing aids was included within the criteria for point 2. The ability to write only one’s signature is considered applicable to meet the criteria for score 1. *Item 5a (Cutting food and handling utensils, without gastrostomy)*: The altered use of cutlery in the context of a functional limitation is explained with examples. Adverbs such as “on occasion” or “on most occasions” indicate the degree of impairment or the need for help. For purposes of harmonizing the complete loss of functionality across all items, score 0 was defined as total dependence with regard to all activities in question. This includes elimination of imprecise descriptions, such as the phrase "must to be fed." *Item 5b (Cutting food and handling utensils, with gastrostomy)*: The extent to which a patient is dependent from a caregiver when handling a feeding tube is specified by score points 1 and 2. *Item 6 (Dressing and hygiene)*: The degree to which assistance or accommodations are needed for certain tasks related to dressing and personal hygiene (such as wearing clothes that are easy to put on and remove, or showering while sitting down) are explained in greater detail.

### Gross motor functions (Items 7–9)

*Item 7 (Turning in bed and adjusting bed clothes)*: The explanation to this item takes into account the gradual transition from independence without increased effort (score 4) to any need for personal assistance (scores 1 and 0). It is also explained how to assess the effort it takes to perform the activity without help (scores 3 and 2). *Item 8 (Walking)* Problems with walking are further described to include issues of unsteadiness and stumbling. The use of walking aids is explicated, and the explanation specifically accounts for patients who must hold onto objects for support. The ability to stand (e.g., for transfer) as a targeted leg movement is introduced as an explanatory example (score 1). *Item 9 (Climbing stairs)* For this item, language is included to account for the necessity of a handrail (score 1) and non-essential use of a handrail (score 2).

### Respiratory function (Items 10–12)

*Item 10 (Dyspnea)*: The phrase "dyspnea and shortness of breath" is consistently used to describe symptoms. Score 3 designates the occurrence of these symptoms during normal walking or moderate (physical) activity. Score 2 symptoms, by contrast, are related to low-intensity activities, which now include “talking for longer periods of time.” The actual use of non-invasive ventilation to treat dyspnea and shortness of breath unambiguously lead to score 0. *Item 11 (Sleep disturbances due to breathing problems, formerly “orthopnea”)*: The explanation for this item specifies that if mechanical ventilation is usually provided, but sleep is still possible without it, breathing should be assessed without the use of ventilation. As the term "orthopnea" does not accurately reflect the spectrum of sleep-related symptoms caused by respiratory impairment, we have chosen to revise the title of this item. Moreover, as reflected in the criteria for score 3, patients may be able to tolerate a flat lateral position while being unable to lie on their back. Sleep disturbances due to respiratory impairment have to be considered most severe when mechanical ventilation is indispensable to achieve acceptable sleep quality (score 0). *Item 12 (Mechanical ventilation)*: Continuous positive airway pressure for treatment of obstructive sleep apnea must not be equated with non-invasive ventilation (NIV). Score 0 is assigned when invasive ventilation procedures are in use. Scores 1–3 are calculated via the amount of NIV use during a 24-h period. If a period equals or exceeds the duration of nighttime sleep but is below permanent dependence (22 h), score 2 is assigned.

## Discussion

We hereby present the ALSFRS-R-SE, a self-explanatory revised scale that has been optimized for use by health care professionals, patients, and caregivers. By adhering as closely as possible to the well-established standardized English version, and by supplementing certain items with explanatory comments and linguistic adaptations, we have attempted to reduce a number of ambiguities which have either developed with several German translations of the ALSFRS-R or are already immanent to the original version of the scale. This multistep process was carried out by a group of experts from 13 German ALS centers, who worked together to both harmonize and sharpen the wording of the ALSFRS-R translation. Through additional specifications, explanations, and examples, it was attempted to reduce the room for interpretation within the original ALS-FRS-R. Professionals from outside the medical field reviewed the ALSFRS-R-SE for comprehensibility and consistency.

With its straightforward language, this self-explanatory version of the ALSFRS-R can be used in a wide range of settings, including in a clinical context with healthcare professionals, or by patients either in a medical venue or remotely.

Only necessary and mostly minor adjustments were made to the original English scale.

Adapting the ALSFRS-R-based remote assessment process requires optimizing both the scale itself and the relevant standard operating procedure [40]. Recent observations by medical professionals indicate that digital platforms and mobile applications have gained substantial acceptance by people with ALS and that their use continues to broaden [6, 41, 42]. However, in order to collect high-quality data via remote digital tools, it is necessary to clearly present and differentiate the questions within the scale.

The suitability as a remote assessment of the ALSFRS-R scale as a primary endpoint in clinical trials (or one of various secondary outcome parameters) would be based on patient-friendliness and scrutinizing the self-explanatory language of the scale. Furthermore, digital instruments-whether computer or smartphone-based-fertilize the ground for remote use of the ALSFRS-R-SE, potentially leading to more frequent assessments and a higher density of ALSFRS-R data [9].

The basis for developing this scale was the fact that the ALSFRS-R has been increasingly used by nurses in the context of regular care as well as by patients themselves, even though the scale was originally devised for health care professionals involved in clinical trials. The expanded use of the scale outside of its original context led to compromises and a reduction of data quality, both issues that have been addressed via the proposed self-explanatory version of ALSFRS-R. We anticipate that this scale will offer additional advantages within clinical trials, as its self-explanatory design reduces the need for rater training.

Despite its clear benefits, the ALSFRS-R has some fundamental limitations that have not been resolved by the current modification and should be highlighted. Sensitivity to disease progression is limited by both the granularity of the items and the frequency of assessment. This limits the ability to depict very rapid and very slow disease progression [43]. While digital self-assessment may increase data density by allowing for more frequent capture [44–46] and while the ALSFRS-R-SE unifies the structure of individual items, there will still be functional changes not covered by the score. Depending on progression rate and phenotype, the sub-scores of the ALSFRS-R will have a different impact on the total score and prognostic models, prompting the suggestion to focus statistical analysis on these sub-scores rather than the total score [26, 47]. Another factor constraining the validity of a self-reported score can be cognitive deficits or affective changes. Identifying such factors is thus essential. However, the ALSFRS-R-SE used by caregivers can be a possible solution to this issue if cognitive deficits are known and recorded.

A clear limitation of the proposed ALSFRS-R-SE is the temporary constraint on the comparability of data gathered from the ALS-FRS-R-SE with those collected using the established English version of the ALSFRS-R. Thus, further studies are needed which apply both versions of the scale in a larger German patient cohort, including evaluation of the ALSFRS-R-SE with regard to its suitability for digital capture and utilization in a mobile application [44, 48]. Despite the uncertainty regarding data comparability, we are convinced that it is justified, and even necessary, to deviate from traditional scoring in order to improve data quality.

## Conclusions

A German consensus group developed an annotated version of the ALSFRS-R scale that is self-explanatory and unambiguous. Given the lack of a standardized German ALSFRS-R, the focus was on creating a German version, but shortcomings of the scale were improved and a qualified English translation was made. The resulting ALSFRS-R-SE can thus be used by healthcare professionals, patients, and their relatives, and is also readily available in a remote setting.

## Supplementary Information

**Additional file 1**: ALSFRS-R-SE German Version.

**Additional file 2**: ALSFRS-R-SE English Version.

## Data Availability

Not applicable.

## Abbreviations

ALSFRS-R: Amyotrophic Lateral Sclerosis Rating Scale, revised
ALSFRS-R-SE: Amyotrophic Lateral Sclerosis Rating Scale, revised, self-explanatory
