# Supplementary material for: ALSFRS-R-SE: an adapted, annotated, and self-explanatory version of the revised amyotrophic lateral sclerosis functional rating scale
Source: Neurol Res Pract. 2022 Dec 15;4:60. doi: 10.1186/s42466-022-00224-6 (PMC9753252; doi:10.1186/s42466-022-00224-6)
Supplement: Supplementary file 1 — Additional file 1: ALSFRS-R-SE German Version. [file 42466_2022_224_MOESM1_ESM.pdf]

ALSFRS-R-SE – selbsterklärende Skala zur Beurteilung der Körperfunktionen bei Amyotropher Lateralsklerose (ALS) *(ALSFRS-R-SE, Amyotrophic Lateral Sclerosis Functional Rating Scale – Revised – self-explanatory)*

Die ALS-Skala, ALSFRS-R-SE, umfasst motorische Funktionsbereiche, die typischerweise durch die ALS eingeschränkt sein können. Die Bewertung kann sowohl von einer anderen Person (z.B. betreuende/r Arzt/Ärztin, Angehörige/r, Pflegepersonal) nach Befragung der an ALS erkrankten Person als auch von dieser selbst durchgeführt werden. Bitte lesen Sie sowohl die Antworten als auch die Erläuterungen sorgfältig durch und bewerten Sie die Funktion bzw. deren ALS-bedingte Einschränkung nach der zum aktuellen Zeitpunkt bestehenden Fähigkeit.

Wenn in einem Funktionsbereich eine Einschränkung vorliegt, die entweder eine andere medizinische Ursache als die ALS hat oder die bereits vor Beginn der ALS bestand (z.B. Einschränkung des Laufens durch eine Hüft-Prothese), kann dieser Bereich als "nicht eingeschränkt" bewertet werden (4 Punkte). Die Funktion sollte immer im Vergleich zu der Zeit vor den ersten ALS-Symptomen beurteilt werden. Es kann von dieser Empfehlung abgewichen werden, wenn Einschränkungen hinzugetreten sind, die wahrscheinlich auf die ALS zurückzuführen sind. Diese Vorgehensweise sollte bei allen Punkten und in zukünftigen ALSFRS-R-Bewertung eingehalten werden.

1. Sprechen

Bitte wählen Sie eine der folgenden Antworten:

(4) ☐ Ungestört
Das Sprechen bzw. die Artikulation ist genauso wie vor dem Auftreten der ersten ALS-Symptome.

(3) ☐ Wahrnehmbare Sprechstörungen
Eine neu aufgetretene Veränderung beim Sprechen, der Artikulation oder der Stimmgebung (Phonation), z. B. im Sinne verwaschenen Sprechens oder einer Heiserkeit, wird entweder selbst oder vom näheren Umfeld bemerkt.

(2) ☐ Wiederholung zur Verständigung notwendig
Es bedarf öfters einer Wiederholung einzelner Worte oder Satzteile, um sich verständlich zu machen.

(1) ☐ Das Sprechen wird mit nonverbaler Kommunikation kombiniert
Schreiben, Verwendung von Kommunikationshilfen oder ähnliche Methoden werden eingesetzt, um das Sprechen zu ergänzen.

(0) ☐ Verlust des verständlichen Sprechens
Der Einsatz von Kommunikationshilfen oder ähnlichen Methoden ist durchgehend notwendig.

2. Speichelfluss

Bitte wählen Sie eine der folgenden Antworten:

(4) ☐ Ungestört
Es sammelt sich kein vermehrter Speichel im Mund.

(3) ☐ Geringfügig, aber eindeutiges Übermaß an Speichel im Mund; nachts Speichelverlust möglich
Vermehrte Speichelansammlung im Mund, jedoch ohne subjektive Beeinträchtigung und ohne Speichelverlust am Tage.

(2) ☐ Mäßig vermehrter Speichelfluss; geringer Speichelverlust möglich
Gelegentlich wird ein Taschentuch verwendet, um tagsüber die Mundwinkel abzutrocknen.

(1) ☐ Deutlich vermehrter Speichelfluss mit etwas Speichelverlust
Speichelverlust ist regelmäßig vorhanden, und ein Taschentuch wird oft, aber nicht immer, verwendet.

(0) ☐ Ausgeprägter Speichelverlust aus dem Mund
Die ständige Verwendung eines Taschentuchs oder einer Absaugung ist erforderlich.

3. Schlucken
Bitte wählen Sie eine der folgenden Antworten:

(4) ☐ Ungestört
Das Schlucken jeglicher Nahrung und von Flüssigkeiten erfolgt ohne Probleme.

(3) ☐ Beginnende Essprobleme mit gelegentlichem Verschlucken
Es wird mehr Zeit zur Nahrungsaufnahme benötigt, oder es werden kleinere Bissen zu sich genommen und mit Vorsicht geschluckt. Es kommt gelegentlich zum Verschlucken oder zu vermehrtem Husten.

(2) ☐ Änderung der Nahrungskonsistenz erforderlich
Die Beschaffenheit von Speisen und Getränken wird verändert oder Schwierigkeiten beim Schlucken führen zur Vermeidung bestimmter Konsistenzen (z. B. Fleisch, trockene Kekse, Nüsse). Ggf. werden wegen einer Schluckstörung ergänzend Zusatznahrung oder Andickungsmittel verwendet.

(1) ☐ Ergänzende Sondenernährung erforderlich
Die Nahrungsaufnahme ist aufgrund der Schluckstörung so schwierig, dass eine Ernährungssonde (PEG) bereits angelegt oder aus ärztlicher Sicht dringend empfohlen wurde, um die Kalorienzufuhr zu ergänzen bzw. die Gefahr des Verschluckens zu vermindern.

(0) ☐ Ernährung ausschließlich über eine Ernährungssonde
Die Aufnahme von Nahrung und Flüssigkeit erfolgt ausschließlich über eine Ernährungssonde. Die orale Nahrungsaufnahme ist aufgrund einer hochgradigen Schluckstörung unmöglich.

4. Handschrift
Bewertet wird das Schreiben mit der Schreibhand in üblicher Haltung.
Bitte wählen Sie eine der folgenden Antworten:

(4) ☐ Ungestört
Das Schreiben mit der üblich verwendeten Schreibhand ist ohne Probleme möglich.

(3) ☐ Langsamer oder unsauber, alle Wörter sind lesbar
Das Schreiben gestaltet sich schwierig oder die Schrift hat sich verändert, selbst wenn die geschriebenen Wörter gut lesbar sind.

(2) ☐ Nicht alle Wörter sind lesbar
Im geschriebenen Text sind einzelne Wörter nicht lesbar. Um lesbar zu schreiben, werden Hilfsmittel wie Schreibhilfen verwendet.

(1) ☐ Das Halten des Stiftes ist möglich, aber nicht das lesbare Schreiben
Der Stift kann gehalten werden. Das Schreiben über den eigenen Namen oder die eigene Unterschrift hinaus ist jedoch nicht möglich.

(0) ☐ Kann Stift nicht halten
Das Halten eines Stiftes, z. B. zum Unterschreiben, ist nicht möglich.

5.a Essen schneiden und Gebrauch von Besteck
Für Personen, die nicht regelhaft eine Ernährungssonde zur Kalorienzufuhr nutzen.
Bitte wählen Sie eine der folgenden Antworten:

(4) ☐ Ungestört
Die Benutzung von Besteck ist ohne Probleme möglich (als Probleme gelten z. B. Messer und Gabel statt Essstäbchen oder die Neigung, vermehrt einen Löffel zu benutzen).

(3) ☐ Etwas langsam und unbeholfen, aber keine Hilfe erforderlich
Aufgrund der Beeinträchtigung der Hände wird mehr Zeit zum Essen benötigt. Die Benutzung von Besteck ist verändert, jedoch selbständig möglich.

(2) ☐ Kann die meisten Speisen schneiden, aber langsam und unbeholfen, braucht teilweise Hilfe
Beim Schneiden bestimmter Speisen wird gelegentlich die Hilfe von einer anderen Person benötigt, oder es werden Hilfsmittel, z. B. in Form von speziellem Besteck, verwendet.

(1) ☐ Essen muss geschnitten werden, kann langsam alleine essen
Für das Schneiden von festen Lebensmitteln ist meistens oder immer eine Unterstützung erforderlich. Selbständiges Essen ist aber möglich (z. B. mit Gabel oder Löffel).

(0) ☐ Essen muss gereicht werden
Nahrung wird immer angereicht. Die selbständige Benutzung von Besteck (z. B. Gabel oder Löffel) ist nicht möglich.

5.b Ernährungssonde und Zubehör handhaben

Für Personen, die regelhaft eine Ernährungssonde zur Kalorienzufuhr nutzen. Bewertet werden soll die vorhandene manuelle Fähigkeit.

Bitte wählen Sie eine der folgenden Antworten, wenn überwiegend eine Ernährungssonde zur Nahrungsaufnahme genutzt wird:

(4) ☐ Ungestört
Die Sonde kann problemlos selbst versorgt und Verschlüsse und Packungen können selbständig geöffnet oder geschlossen werden.

(3) ☐ Etwas langsam und unbeholfen, aber alle Handgriffe können selbständig ausgeführt werden
Es wird keine Unterstützung bei der Handhabung der Ernährungssonde benötigt, allerdings ist der Umgang erschwert.

(2) ☐ Beim Umgang mit Verschlüssen und Deckeln teilweise Hilfe erforderlich
Der Umgang mit der Sonde ist überwiegend selbständig möglich. Hilfe wird jedoch z. B. beim Öffnen von Verschlüssen benötigt.

(1) ☐ Minimale Unterstützung kann bei der Sondenversorgung gegeben werden
Die Versorgung der Ernährungssonde muss überwiegend von einer anderen Person übernommen werden. Minimale Handgriffe können selbständig getätigt werden.

(0) ☐ Kann an keiner Stelle bei der Sondenversorgung mithelfen
Die Versorgung der Ernährungssonde muss vollständig von einer anderen Person übernommen werden. Es können keine Handgriffe selbst getätigt werden.

6. Ankleiden und Körperpflege

Bitte wählen Sie eine der folgenden Antworten:

(4) ☐ Ungestört
Das Ankleiden und die Körperpflege sind ohne Probleme möglich.

(3) ☐ Keine Hilfe erforderlich, jedoch mit Mühe verbunden
Das Ankleiden oder die Körperpflege sind langsamer als zuvor, bleiben jedoch unabhängig und ohne Unterstützung durch Hilfsverfahren oder durch eine andere Person möglich.

(2) ☐ Zeitweilig sind Hilfe oder Hilfsverfahren erforderlich
Es wird gelegentlich Hilfe durch eine Person in Anspruch genommen, oder es werden Strategien entwickelt, um Beeinträchtigungen auszugleichen (z. B. leicht an- und ausziehbare Kleidung, Sitzen beim Ankleiden oder Duschen, Nutzung von Hilfsmitteln).

(1) ☐ Hilfe durch andere Personen erforderlich
Beim Ankleiden und der Körperpflege ist regelmäßig die Unterstützung durch eine andere Person notwendig.

(0) ☐ Vollständige Abhängigkeit
Das Ankleiden und die Körperpflege müssen vollständig von einer anderen Person übernommen werden.

7. Umdrehen im Bett und Richten der Bettdecke

Bitte wählen Sie eine der folgenden Antworten:

(4) ☐ Ungestört
Das Umdrehen im Bett und Richten der Bettdecke lassen sich ohne Probleme durchführen.

(3) ☐ Etwas langsam und unbeholfen, aber keine Hilfe erforderlich
Es ist schwieriger, sich im Bett umzudrehen oder die Bettdecke zu richten.

(2) ☐ Selbständig möglich, jedoch mit großer Mühe verbunden
Das Umdrehen im Bett und das Richten der Bettdecke sind nur mit großer Mühe möglich. Eventuell wird eine der beiden Tätigkeiten unterstützt oder es wird zum Umdrehen ein Haltegriff verwendet.

(1) ☐ Kann zum Umdrehen oder Richten der Bettdecke ansetzen, aber die Bewegung nicht selbständig vollenden
Das Drehen im Bett oder Richten der Bettdecke kann begonnen werden. Es wird jedoch Unterstützung durch eine Person oder Hilfsmittel benötigt, um die jeweilige Tätigkeit zu vollenden.

(0) ☐ Vollständige Abhängigkeit
Beim Umdrehen im Bett und dem Richten der Bettdecke ist durchgehend Hilfe erforderlich

8. Gehen

Bitte wählen Sie eine der folgenden Antworten:

(4) ☐ Ungestört
Es besteht keine Veränderung der Gehfähigkeit.

(3) ☐ Beginnende Gehschwierigkeiten
Es zeigen sich Veränderungen, die eine Verlangsamung, Unsicherheit oder Stolpern einschließen. Hilfe in Form einer anderen Person, durch Festhalten an Gegenständen oder durch die Verwendung von Gehhilfen außerhalb des Wohnbereichs (z. B. Fußheberschiene, Gehstock, Rollator) wird jedoch nicht regelhaft benötigt.

(2) ☐ Gehen nur mit Unterstützung möglich
Das Gehen erfordert regelhaft ein Festhalten oder - außerhalb des Wohnbereichs - Hilfe durch Fußheberschienen, Gehhilfen oder durch eine andere Person.

(1) ☐ Nicht gehfähig, aber gezielte Bewegungen der Beine möglich
Die Beine können gezielt bewegt werden. Das Stehen mit Unterstützung, z. B. zum Transfer, kann möglich sein. Auch mit Unterstützung anderer Personen oder Gehhilfen besteht keine Gehfähigkeit.

(0) ☐ Keine zielgerichtete Beinbewegung möglich
Die Beine können das Körpergewicht nicht tragen (z. B. zum Transfer), und es können keine zweckdienlichen Beinbewegungen z. B. zur Erleichterung der Pflege ausgeführt werden.

9. Treppen hinaufsteigen

Bitte wählen Sie eine der folgenden Antworten:

(4) ☐ Ungestört
Es besteht keine Veränderung beim Hinaufsteigen einer Treppe.

(3) ☐ Langsam
Das Hinaufsteigen einer Treppe erfolgt verlangsamt, ist aber ohne Ruhepausen oder Unsicherheit möglich.

(2) ☐ Leichte Unsicherheit oder Ermüdung
Das Hinaufsteigen einer Treppe ist unsicherer. Eventuell sind Ruhepausen nötig. Hilfe, z. B. durch einen Handlauf oder eine andere Person, ist nicht zwingend erforderlich.

(1) ☐ Unterstützung erforderlich
Das Hinaufsteigen einer Treppe ist ohne die Verwendung eines Handlaufs oder die Hilfe einer Person nicht möglich.

(0) ☐ Nicht möglich
Treppen können auch mit Hilfe nicht hinaufgestiegen werden.

10. Luftnot und Kurzatmigkeit

Bitte wählen Sie eine der folgenden Antworten:

(4) ☐ Ungestört
Luftnot oder Kurzatmigkeit tritt bei üblicher Belastung im Alltag nicht auf.

(3) ☐ Beim Gehen
Luftnot oder Kurzatmigkeit tritt beim Gehen in Schrittgeschwindigkeit oder bei mäßiger Belastung auf.

(2) ☐ Auftreten bei Aktivitäten des täglichen Lebens einschließlich Essen, Baden, Ankleiden
Luftnot oder Kurzatmigkeit tritt bei geringer Belastung oder längerem Sprechen auf.

(1) ☐ Auftreten in Ruhe, im Sitzen oder im Liegen
Luftnot oder Kurzatmigkeit besteht ohne Belastung entweder im Sitzen oder im Liegen oder in beiden Positionen.

(0) ☐ Erhebliche Luftnot oder Kurzatmigkeit in Ruhe. Eine mechanische Beatmung wird aufgrund der Luftnot oder einer Kurzatmigkeit genutzt oder empfohlen.
Luftnot oder Kurzatmigkeit ist in Ruhe immer deutlich vorhanden und/oder eine Maskenbeatmung (nicht-invasive Beatmung) oder Beatmung über Luftröhrenschnitt (Tracheostoma) muss zur Linderung von Luftnot und Kurzatmigkeit eingesetzt werden.

11. Atmungsbedingte Schlafstörung

Wenn eine nächtliche Beatmung vorgesehen ist, das Schlafen jedoch ohne diese erfolgt, soll die nächtliche Atmung ohne die Nutzung der Beatmung beurteilt werden.

Bitte wählen Sie eine der folgenden Antworten:

(4) ☐ Ungestört
Das Ein- und Durchschlafen ist nicht durch Luftnot oder Kurzatmigkeit beeinträchtigt.

(3) ☐ Wiederholte Schlafstörung durch Luftnot oder Kurzatmigkeit, keine Verwendung von mehr als zwei Kissen
Es bestehen bereits beim Hinlegen oder während der Nacht Luftnot oder Kurzatmigkeit. Das Schlafen auf der Seite kann die Atmung verbessern. Der Oberkörper wird nicht durch mehr als zwei Kopfkissen oder mittels eines um mehr als 30 Grad hochgestellten Kopfteils aufgerichtet.

(2) ☐ Regelmäßige Verwendung von mehr als zwei Kissen zum Schlafen erforderlich
In flacher Rückenlage ist das Atmen merklich unangenehm und das Ein- oder Durchschlafen dadurch gestört. Der Oberkörper wird durch drei oder mehr Kissen oder mittels eines um mehr als 30 Grad hochgestellten Kopfteils aufgerichtet.

(1) ☐ Schlafen nur im Sitzen möglich
Zum Schlafen ist eine Sitzposition entweder im Bett oder in einem Stuhl notwendig.

(0) ☐ Hochgradige Schlafstörung aufgrund von Luftnot oder Kurzatmigkeit
Aufgrund von Luftnot oder Kurzatmigkeit ist das Schlafen ohne Maskenbeatmung (nicht-invasive Beatmung) oder Beatmung über Luftröhrenschnitt (Tracheostoma) nicht möglich. Eine mechanische Beatmung wird zur Beschwerdelinderung regelhaft genutzt.

12. Mechanische Beatmung

Bitte wählen Sie eine der folgenden Antworten:

(4) ☐ Nicht erforderlich
Die Atmung erfolgt immer selbständig ohne die Nutzung einer mechanischen Beatmung. Eine nächtliche Druckunterstützung (z. B. CPAP-Therapie zur Behandlung eines Schlafapnoe-Syndroms) zählt nicht als mechanische Beatmung.

(3) ☐ Zeitweilige Nutzung einer nicht-invasiven Beatmung
Eine Maskenbeatmung (nicht-invasive Beatmung, z. B. BiPAP) wird unregelmäßig oder für weniger als die Dauer des Nachtschlafs eingesetzt.

(2) ☐ Ständige Nutzung einer nicht-invasiven Beatmung in der Nacht oder zusätzlich stundenweise am Tag
Eine Maskenbeatmung (nicht-invasive Beatmung) wird regelmäßig während der Nacht oder zusätzlich stundenweise während des Tages eingesetzt (insgesamt 8 bis 22 Stunden täglich bezogen auf 24 Stunden).

(1) ☐ Kontinuierliche Nutzung einer nicht-invasiven Beatmung (Tag und Nacht)
Eine Maskenbeatmung (nicht-invasive Beatmung) wird nahezu die gesamte Zeit eingesetzt (mehr als 22 Stunden täglich bezogen auf 24 Stunden).

(0) ☐ Mechanische Beatmung über Intubation oder Luftröhrenschnitt (Tracheostoma)
Es erfolgt eine kontinuierliche mechanische Beatmung über einen Beatmungsschlauch (Intubation) oder einen Luftröhrenschnitt (Tracheostoma).

Informationen zur konsentierten Version ALSFRS-R-SE

Der vorliegende ALSFRS-R-SE basiert auf einem gemeinsam gefassten Konsens im ALS-Forschungsnetzwerk MND-NET. Die Funktionsbereiche entsprechen der englischen Originalversion des ALSFRS-R (Cedarbaum et al. 1996) und wurden in Deutsch übersetzt. Die konsentierte Version des ALSFRS-R-SE enthält einen Einleitungstext, allgemeinverständliche Sprache und erläuternde Kommentare zu den einzelnen Auswahlmöglichkeiten. Durch den selbsterklärenden Charakter ist der Einsatz dieser Version sowohl bei Fremdbewertungen als auch bei Selbstbewertung durch Personen, die selbst von ALS betroffen sind, möglich.

Datum der letzten Überarbeitung: 07/2022

Lizenz: Die Verwendung der konsentierten Fassung des ALSFRS-R-SE ist ohne Lizenz möglich.

Kontakt: Dr. André Maier, Ambulanz für ALS und andere Motoneuronenerkrankungen der Charité, Universitätsmedizin Berlin; andre.maier@charite.de
