# Supplementary material for: ALSFRS-R-SE: an adapted, annotated, and self-explanatory version of the revised amyotrophic lateral sclerosis functional rating scale
Source: Neurol Res Pract. 2022 Dec 15;4:60. doi: 10.1186/s42466-022-00224-6 (PMC9753252; doi:10.1186/s42466-022-00224-6)
Supplement: Supplementary file 2 — Additional file 2: ALSFRS-R-SE English Version. [file 42466_2022_224_MOESM2_ESM.pdf]

ALSFRS-R-SE – Physical function assessment scale in amyotrophic lateral sclerosis (ALS)
(ALSFRS-R-SE, Amyotrophic Lateral Sclerosis Functional Rating Scale – Revised – self-explanatory)

The self-explanatory Amyotrophic Lateral Sclerosis Functional Rating Scale – Revised (ALSFRS-R-SE) is comprised of various motor functioning items that typically have limiting characteristics in ALS. The ALSFRS-R-SE assessment can be performed by patients themselves as well as by others (e.g., an attending physician, a relative, a healthcare professional) following an interview with the affected. Please carefully read the explanations and options and provide an assessment of functionality, and, respectively, relevant ALS-related limitations that reflect actual capacities at the time of filling in the questionnaire.

If the cause of a limitation in any functional area is attributable to a medical condition other than ALS, or if a limitation was already present before the onset of ALS (e.g., gait impairment following hip replacement surgery) the respective item can be assessed as "normal" (4 score points). Functionality should always be assessed relative to one's status before the onset of initial ALS symptoms. The affected may deviate from this recommendation if they are experiencing additional limitations that are likely to be due to ALS. Once an approach is chosen, please be consistent in following it when answering all questions on this and on future ALSFRS-R-SE assessments.

1. Speech
Please choose one of the following answers:

(4) ☐ Normal
Speaking and/or articulation is the same as it was before the onset of initial ALS symptoms.

(3) ☐ Detectable speech disturbance
Speech, articulation or phonation have changed, as perceived either by the affected themselves or by their immediate contacts. This may present as slurred speech or hoarseness of voice.

(2) ☐ Intelligible speech with repetition
Frequent repetition of single words or parts of a sentence are required to convey meaning.

(1) ☐ Speech combined with non-vocal communication
Writing things down, use of communication aids, and similar methods are needed to convey meaning.

(0) ☐ Loss of useful speech
Use of communication aids or similar methods are always required.

2. Salivation
Please choose one of the following answers:

(4) ☐ Normal
No excess saliva accumulates in the mouth.

(3) ☐ Slight but definite excess of saliva in mouth; night-time drooling may take place
Increased accumulation of saliva in the mouth; however, subjectively not an impairment or impediment and no loss of saliva during the day.

(2) ☐ Moderately excessive saliva; may experience minimal drooling
During the day, a tissue is occasionally used to dab the edges of the mouth.

(1) ☐ Marked excess of saliva with some drooling
Regular loss of saliva, a tissue is used often but not constantly.

(0) ☐ Marked drooling
Permanent use of tissues or a suction device is required.

3. Swallowing
Please choose one of the following answers:

(4) ☐ Normal
Swallowing any type of food or liquid is unproblematic.

(3) ☐ Minor swallowing problems - occasional choking
Food intake takes longer; food must be cut into smaller bites and swallowed with care. Occasionally, choking on food or higher frequency of coughing is observed.

(2) ☐ Dietary consistency changes
Difficulty swallowing (dysphagia) and trouble with certain consistencies of food and beverages results in the avoidance of some types of food consistencies (e.g., meat, dry biscuits, nuts). Dietary supplements or thickeners may be used due to difficulty in swallowing.

(1) ☐ Supplemental tube feeding.
Due to dysphagia, food intake has become so difficult that an enteral feeding tube (PEG) must be fitted or is highly recommended by the physician to supplement caloric intake and/or prevent choking on food.

(0) ☐ Exclusively enteral tube feeding
Food and liquid intake happen exclusively via a feeding tube; oral food intake is impossible due to high-grade dysphagia.

4. Handwriting
The subject of this assessment is writing with one's dominant hand (writing hand) in the usual posture.
Please choose one of the following answers:

(4) ☐ Normal
Writing with the dominant writing hand causes no problems.

(3) ☐ Slow or sloppy but all words are legible
Writing is more difficult, or alternately, the appearance of a person's written text has changed even though the words remain legible.

(2) ☐ Not all words are legible
Some written words are illegible. Writing aids are used to promote legibility.

(1) ☐ No words are legible, but can still grip pen
Holding a pen is possible; however, anything beyond signing or writing one's own name is not.

(0) ☐ Unable to grip pen
Holding a pen is impossible.

5.a Cutting food and handling utensils
Pertaining to persons <u>not</u> regularly using an enteral feeding tube for caloric intake.
Please choose one of the following answers:

(4) ☐ Normal
The use of cutlery is not problematic. Problems would be, for example, the use of knives and forks instead of chopsticks or the inclination to use a spoon more often.

(3) ☐ Somewhat slow and clumsy, but no help needed
Eating takes more time due to impairment of the hands. Method of handing cutlery has changed, but its use is still possible without assistance.

(2) ☐ Can cut most foods, although slowly and clumsily; some help is needed
Assistance is needed on occasion when cutting certain types of foods; alternately, eating aids such as special cutlery are in use.

(1) ☐ Food must be cut by someone else, but can still feed themself slowly
Assistance is needed to cut solid food and on most other occasions. However, eating on one's own is still possible (e.g., using a fork or a spoon).

(0) ☐ Total dependence
The affected person is unable to use cutlery (e.g., a fork or a spoon) on their own, and can only eat when fed.

5.b Cutting food and handling utensils

For persons regularly in need of a feeding tube for caloric intake. The subject of this assessment is manual dexterity.

Please choose one of the following answers if food intake is predominantly conducted via a feeding tube:

(4) ☐ Normal
The tube can be handled autonomously, and locks and packets can be opened and closed without assistance.

(3) ☐ Clumsy but able to perform all manipulations independently
No assistance is needed when handling the feeding tube, however use is somewhat difficult.

(2) ☐ Some help needed with closures and fasteners
Handling the feeding tube is done more or less independently. Assistance is needed when opening locks and fasteners.

(1) ☐ Provides minimal assistance to caregiver
Another person mostly handles the feeding tube. The affected can only carry out minimal actions themselves.

(0) ☐ Total dependence
Handling the feeding tube is done entirely by another person. No actions can be executed by the affected.

6. Dressing and hygiene

Please choose one of the following answers:

(4) ☐ Normal
Getting dressed and tending to personal hygiene are unproblematic.

(3) ☐ Independent and complete self-care requires effort and is less efficient
Getting (un)dressed and tending to personal hygiene are executed more slowly than before but are performed autonomously and require neither aids nor assistance from another person.

(2) ☐ Intermittent assistance or substitute methods
At times, another person is called upon to assist, or strategies are developed to counteract impairment (e.g., wearing clothes that are easy to put on or take off, getting (un)dressed or showering while sitting down, use of aids).

(1) ☐ Needs attendant for self-care
Another person is required on a regular basis to (un)dress and attend to the affected person’s personal hygiene.

(0) ☐ Total dependence
Dressing, undressing and personal hygiene must be entirely performed by another person.

7. Turning in bed and adjusting bed clothes

Please choose one of the following answers:

(4) ☐ Normal
Turning in bed and handling blankets do not cause problems.

(3) ☐ Somewhat slow and clumsy, but no help needed
Turning in bed or handling blankets is difficult.

(2) ☐ Can turn on their own and adjust sheets, but with great difficulty
Turning in bed and handling blankets is possible but requires great effort. Either action may require support, or a grip may be used when turning in bed.

(1) ☐ Can initiate action, but not turn or adjust sheets without assistance
The actions of turning in bed and handling blankets can be initiated, however another person's assistance is required to complete these actions.

(0) ☐ Total dependence
Assistance is consistently required when turning in bed or handling blankets.

8. Walking
Please choose one of the following answers:

(4) ☐ Normal
No change in walking ability.

(3) ☐ Minor ambulation difficulties
Changes, such as walking more slowly, stumbling, or a loss of stability, are apparent, although the affected does not require outside assistance on a regular basis, either in the form of another person, a walking aid (e.g., foot lifter, cane, walkers) or holding on to a stable object.

(2) ☐ Walks with assistance
The affected regularly requires assistance when walking — either in the form of holding on to something, or, outside the home, use of a foot lifter, walking aids or help from another person.

(1) ☐ Nonambulatory functional movement
Targeted leg movements are still possible. Standing with support, e.g., for transfer, can be possible. The affected has no ambulatory capacity, not even with the assistance of another person.

(0) ☐ No purposeful leg movement
The legs cannot support the weight of the body (e.g., for transfer), no purposeful movements can be executed, such as helping with care activities.

9. Climbing the stairs
Please choose one of the following answers:

(4) ☐ Normal
No change is observed when climbing the stairs.

(3) ☐ Slow
Climbing the stairs without taking a break or feeling unstable is possible if done slowly.

(2) ☐ Mild unsteadiness or fatigue
Climbing the stairs is accompanied by a feeling of instability, and breaks might be necessary. Use of a handrail or assistance from another person are not absolutely necessary.

(1) ☐ Needs assistance
Climbing the stairs cannot be executed without use of a handrail or assistance from another person.

(0) ☐ Cannot do
Stairs cannot be climbed, even with assistance or support.

10. Dyspnea and shortness of breath
Please choose one of the following answers:

(4) ☐ None
No dyspnea or shortness of breath when performing daily routines at normal intensity.

(3) ☐ Occurs when walking
Dyspnea or shortness of breath may occur when walking at a normal pace or performing activities at moderate intensity.

(2) ☐ Occurs with one or more of the following: eating, bathing, dressing (ADL)
Dyspnea or shortness of breath may occur when performing activities at low intensity or when talking for longer periods of time.

(1) ☐ Difficulty breathing when at rest, including either sitting or lying down
Dyspnea or shortness of breath in the absence of any physical strain when either sitting and/or lying down.

(0) ☐ Significant difficulty breathing, mechanical respiratory support may be needed
Significant dyspnea or shortness of breath is present when at rest; mask ventilation (non-invasive ventilation) or ventilation via tracheostomy must be applied to alleviate dyspnea and shortness of breath.

11. Sleep disturbance due to breathing problems

If mechanical ventilation is usually provided during the night, but sleep is possible without it, nighttime breathing should be assessed without the use of ventilation.

Please choose one of the following answers:

(4) ☐ None
Falling asleep and sleeping through the night are unimpaired by dyspnea or shortness of breath.

(3) ☐ Some difficulty sleeping at night due to shortness of breath, more than two pillows are not routinely used
Dyspnea and shortness of breath are present at night and when lying down. Breathing may be improved by sleeping on one side. To support the torso, a maximum of two pillows are used or the head section of the bed may be elevated by no more than 30 degrees.

(2) ☐ More than two pillows are needed in order to sleep
When lying down flat on one's back, breathing is noticeably bothersome, which in turn disturbs the process of falling asleep and sleeping through the night. To support the torso, three or more pillows are used or the head section of the bed is elevated by more than 30 degrees.

(1) ☐ Can only sleep sitting up
A seated position must be assumed, either in bed or on a chair, to sleep.

(0) ☐ Unable to sleep
Due to dyspnea or shortness of breath, sleep is impossible without mask ventilation (non-invasive ventilation) or ventilation via tracheostomy. Mechanical ventilation is in regular use to alleviate symptoms.

12. Mechanical ventilation

Please choose one of the following answers:

(4) ☐ None
Breathing is always an autonomous action, not requiring use of mechanical ventilation. Nocturnal air pressure support (i.e., CPAP therapy to treat sleep apnea syndrome) does not constitute mechanical ventilation.

(3) ☐ Intermittent use of non-invasive ventilation
Mask ventilation (non-invasive ventilation, e.g., BiPAP) is in use at irregular intervals or for a shorter period of time than the normal nocturnal sleep cycle.

(2) ☐ Continuous use of non-invasive ventilation during the night
Mask ventilation (non-invasive ventilation) is in regular use at night and possibly on an hourly basis during the day (a total of 8 to 22 hours in any 24-hour cycle).

(1) ☐ Continuous use of non-invasive ventilation during the night and day
Mask ventilation (non-invasive ventilation) is in use almost all of the time (more than 22 hours in any 24-hour cycle).

(0) ☐ Invasive mechanical ventilation by intubation or tracheostomy
Continuous mechanical ventilation via a ventilation tube (intubation) or tracheostomy.
